# A Quality Improvement Initiative to Reduce Duplicate Inflammatory Marker Use

**DOI:** 10.1097/pq9.0000000000000769

**Published:** 2024-09-19

**Authors:** Kathryn E. Bakkum, Kathy H. Stoner, David A. Gannon, Thomas B. Mike, Prabi Rajbhandari

**Affiliations:** From the * Division of Hospital Medicine, Department of Pediatrics, Akron Children’s Hospital, Akron, Ohio; † Department of Quality Services, Akron Children’s Hospital, Akron, Ohio; ‡ Lab Administrative Office, Akron Children’s Hospital, Akron, Ohio.

## Abstract

**Introduction:**

Inflammatory markers (IMs) are often ordered in multiples, even though evidence suggests that this does not add any clinical benefit. The project aimed to reduce the number of duplicate IMs for patients by 10% in 12 months.

**Methods:**

We implemented a quality improvement (QI) project at our hospital, focusing on patients admitted to the pediatric hospital medicine service. The team chose the model for improvement as the QI methodology. Key interventions included ongoing provider education, integrating the project into the physician incentive plan, and reviewing disease-specific pathways. The primary outcome measure was “duplicate IM use,” which was defined as any two or more IMs (procalcitonin, C-reactive protein, or erythrocyte sedimentation rate) obtained on the same patient within 24 hours. The secondary outcome measure was any IM use during their stay, and the balancing measures were average complete blood count use, hospital length of stay, and 7-day readmission rate.

**Results:**

The baseline duplicate IM use, and any IM use was 43% and 19%, respectively. After the start of this QI project, duplicate IM use decreased to 12%, and the use of any IM also decreased to 12%. Complete blood count use varied from 11% to 24% during the project without obvious correlation to IM use. Hospital length of stay decreased from 2.5 to 2.6 days, and the 7-day readmission rate remained at 2.8%.

**Conclusions:**

The duplicate IM use and IM use were decreased without a concurrent increase in the balancing measures, indicating that a safe reduction of IM testing is feasible in inpatient pediatric care.

## INTRODUCTION

Low-value care is one of the six domains of healthcare waste that accounts for 25% of healthcare expenditure in the United States.^1^ A 15-year meta-analysis estimated that 21% of laboratory tests are ordered unnecessarily, and the risk of overutilization is six times more likely during the initial diagnostic evaluation.^2^ Although these unnecessary laboratory results do not offer any additional benefit, they can result in additional testing and unnecessary procedures, ultimately increasing patient costs.^3^

Inflammatory markers (IMs)—C-reactive protein (CRP), procalcitonin (PCAL), and erythrocyte sedimentation rate (ESR)—are commonly used tests that are often ordered concurrently for the evaluation of infection, despite evidence suggesting that the simultaneous use of multiple IMs increases costs without improving patient care.^4^ Compared with CRP and ESR, PCAL is relatively new, and its exact utility in pediatrics is still being explored. PCAL has a better positive predictive value than CRP for febrile infants less than 2 months^5,6^ and in the diagnosis of sepsis.^7^ PCAL may help exclude typical bacterial pneumonia,^8^ but variability between studies suggests an inconsistent ability to distinguish between viral and bacterial etiologies reliably.^9,10^ Although there is good evidence to support PCAL-guided de-escalation of antibiotics in sepsis,^11^ it is essential for providers to consider various factors such as site of infection, microbial etiology, and the patient’s response to initial treatment. The impact of IM testing on outcomes such as length of stay (LOS) and readmission varies, suggesting that this is an area for additional investigation and a possible focus for quality initiatives to improve evidence-based practices.^12,13^

At our institution, multiple IMs are commonly obtained during a patient’s hospitalization. Because PCAL was relatively new to our practice and introduced without guidelines, some team members felt less comfortable relying solely on PCAL and consequently would supplement their evaluation by adding CRP. Providers also obtained PCAL for disease processes, such as gastroenteritis or cellulitis, for which its clinical application is not established.^14,15^ In instances of elevated PCAL, providers may encounter uncertainty, potentially resulting in prolonged hospitalization as they monitor the level over time until it reaches a “safe” level arbitrarily determined by the provider.

To address the lack of high-value care related to IMs, a quality improvement (QI) project was started in January 2022. The goal was to decrease duplicate IM testing for hospitalized patients admitted to the pediatric hospital medicine (PHM) service from 43% to 33%, representing a 10% absolute reduction rate in 12 months.

## METHODS

### Context

Our institution is an independent, free-standing children’s hospital with 449 beds that admits more than 10,000 patients yearly. The PHM service admits more than 4,000 patients annually and is the largest hospital admitting service. PHM admits children with general pediatric conditions and also oversees the care of medically complex patients. Patients are admitted to one of the three age-based units (infant, school-aged, and adolescent), with the transitional care unit reserved for patients with chronic complex conditions requiring long-term management. The PHM service comprises two resident teams and one advanced practice provider team with physician oversight. Resident teams are comprised of two to five trainees staffed by an attending and sometimes accompanied by a PHM fellow. The advanced practice provider team consists of two to three nurse practitioners who oversee consults, surgical co-management, and general pediatric admissions.

The model for improvement, developed by the Institute for Healthcare Improvement, was utilized as the QI methodology for the study.^16^ The hospital’s institutional review board determined this study was QI work and exempt from further institutional review board review.

### Interventions

**Fig. 1.**
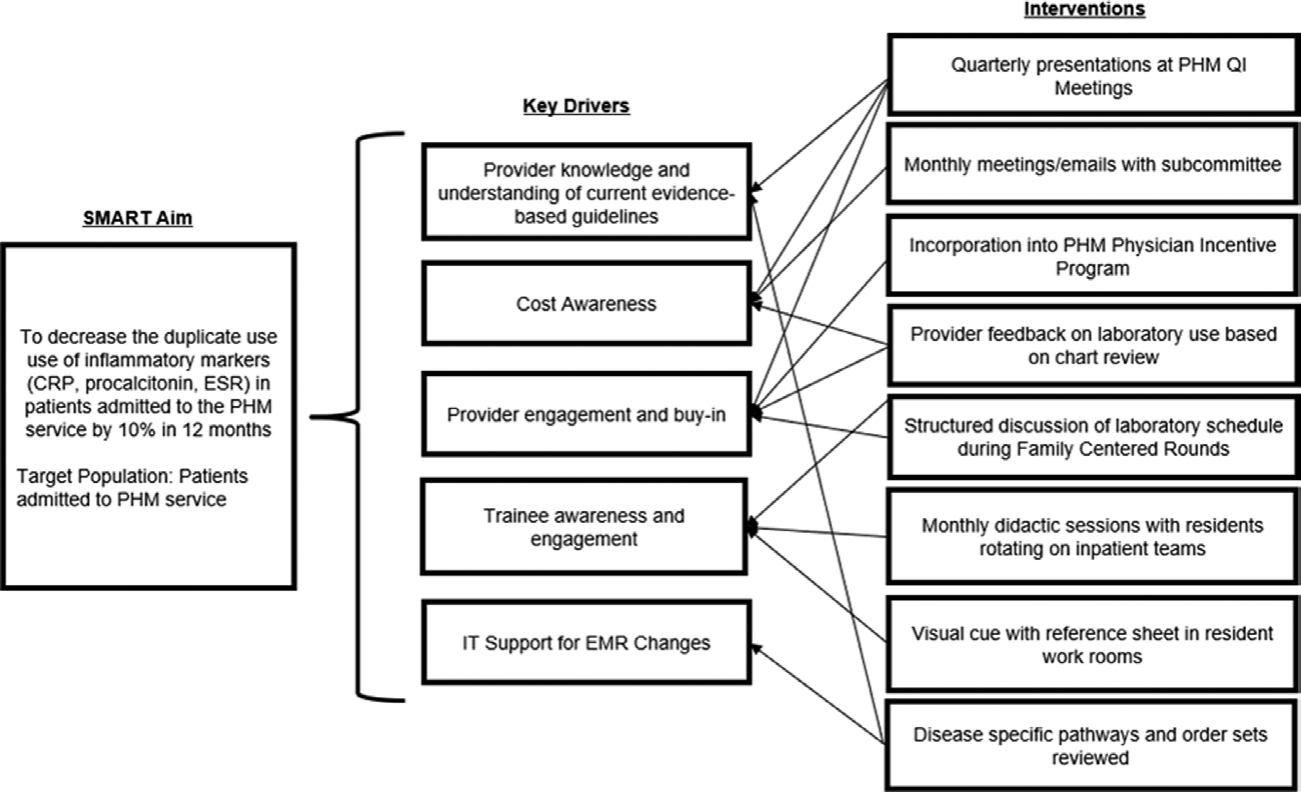
Key driver diagram. CRP, ESR, PHM, IT, EMR, QI. EMR, electronic medical record; IT, information technology.

We formed a multidisciplinary QI team consisting of six hospitalists, one PHM fellow, a clinical pathologist, a laboratory supervisor, a data analyst, and a QI specialist. After an initial meeting between team members, they created a key driver diagram and identified potential interventions (Fig. 1).

#### Stakeholder Buy-in and Project Awareness

Before initiating this project, the project was presented to the hospital-based cluster QI committee, clinical effectiveness committee, order set committee, resident leadership, and chief quality officer, along with the directors and chairs of the Department of Pediatrics and Pathology. It was then presented to division members to garner their support. During this meeting, the project rationale and baseline data were explained, and an early version of the key driver diagram was shared to allow input regarding proposed interventions.

#### Adding the Project to Physician Incentive Plans

At our institution, each division typically selects one to three QI projects annually as physician incentive plan (PIP) projects, which offer incentive payments to providers for meeting specific metrics. This project was adopted by the PHM division as one of the PIP projects to reduce duplicate IM use by 10% by the division and maintain that reduction over time.

#### Provider Education and Feedback

After obtaining buy-in from the division members, the team conducted an educational session for all hospitalists. This session included a review of the pathophysiology and kinetics of various IMs. The educational sessions also included cost information for the tests and a brief review of institution-based guidelines where PCAL or CRP was recommended. Subsequent divisional project updates occurred quarterly and periodically via email.

To help spread the information to residents, the team created an education slide deck for hospitalists who round on the resident teams. The six hospitalists involved with the project were primarily responsible for these education sessions. Educational sessions were started in March 2022 and beginning in August 2022, we developed a calendar to ensure that a minimum of one education session was provided to each resident team during every rotation block.

A reference sheet detailing the pathophysiology and kinetics of IMs was also developed. Digital copies were emailed to hospitalists and pediatric residents. Hard copies were also created and placed in the resident workrooms for easy access.

#### Structured Laboratory Discussion During Family-centered Rounds

A separate initiative to improve the quality of family-centered rounds (FCRs) was already in process at our institution. The FCR team developed a checklist including one item for “Order Readback.” The checklist was used to remind the FCR team to discuss upcoming laboratory orders and determine if laboratories were still indicated. This concept was initially introduced at the resident house staff meeting and then reinforced as part of monthly resident education.

#### Clinical Pathway and Order Set Review

All inpatient institutional clinical pathways and order sets were reviewed. Order sets are a group of standard orders (laboratory results, medications, instructions, etc.) specific to a disease, which were created within Epic, the electronic medical software used by our institution.^17^ At our institution, PHM owns 49 order sets, of which 14 included CRP or PCAL as part of the order set. ESR was not included in any order sets. The labs were never preselected, leaving it to the provider to decide when the tests were needed. During the project, two changes were made. First, there was an update to the order set for evaluating well-appearing febrile infants. The order set and pathway were changed to state that PCAL is preferred and is the only IM included in the order set. The second change occurred within the order set for community-acquired pneumonia. The order set previously contained both CRP and PCAL. CRP was removed from the order set in August 2022. Other pathways and order sets were reviewed for their compliance with current evidence-based guidelines, and no further changes were necessary.

### Measures

Measures are outlined in detail in Table 1. The primary outcome measure was the weekly percentage of duplicate IMs obtained for patients admitted to the PHM service. The numerator was the number of patients per week with duplicate IMs. The denominator was all patients admitted per week to the PHM service who had at least one IM obtained during their hospital stay. The key concept behind our initiative was to ensure that IMs were not repeated unnecessarily within a short timeframe. “Duplicate IM” was defined as using any combination of two or more IMs (CRP, PCAL, or ESR) obtained within 24 hours during admission. For example, two PCALs resulted 6 hours apart, or one PCAL and one CRP resulted simultaneously. Laboratory results obtained in the emergency department were not included in this study as the interventions focused solely on PHM service. Data were obtained from SAP BusinessObjects, a browser-based query and reporting tool that communicates with Epic.^18^ The accuracy and quality of data were validated by two team members (K.E.B. and D.A.G.) by manually reviewing all charts for the first four cycles and periodic review of a selected subset of charts throughout the project. The secondary measure was the use of any IM during a patient’s stay, calculated by the weekly percentage of patients with at least one or more IM during their admission. The process measure was the percentage of completed monthly educational sessions provided to the residents. The balancing measures were the average hospital LOS, 7-day readmission rate (obtained via our division dashboard), and the weekly percentage of complete blood count (CBC) used on the PHM service, obtained using the same query as IM. The weekly rate of CBC use was chosen to assess if providers were trending CBC instead of using IMs. The measures were tracked using run charts or statistical process control charts as appropriate. Standard rules to identify special cause variation were utilized, specifically eight consecutive points on either side of the mean.^19^

**Table 1.** Operational Definitions of Key Project Measures

Measurement Group	Measure	Numerator/Denominator
Primary outcome measure	Duplicate inflammatory marker use	Numerator = the number of patients per week on whom two or more IM (CRP, PCAL, or ESR, in any combination) were resulted within a 24-h period Denominator = all patients admitted per week to PHM service who had at least one IM obtained
Secondary outcome measure	Any inflammatory marker use	Numerator = the number of patients per week who had at least one inflammatory marker obtained Denominator = all patients admitted per week to PHM service
Process measure	Educational sessions	Numerator = the number of educational sessions with residents Denominator = planned 10 scheduled sessions
Balancing measures	Readmission rate	Numerator = the number of unplanned readmissions within 7 d Denominator = all patients admitted to the PHM service per month
	CBC use	Numerator = all patients admitted to PHM service with at least 1 IM obtained who also had a CBC Denominator = all patients admitted per week to PHM service
	LOS	Numerator = the total number of patient days per month spent admitted to PHM Denominator = the total number of patients per month admitted to PHM service

To measure the financial impact of testing on patient-level billing, charges for IM testing were calculated, expressed as weekly charges per patient. The charge data were not actively measured during the project. However, upon the project’s conclusion, the finance department was retrospectively requested to calculate the charges which were measured during the pre- and postintervention phase. This was calculated by using the patient charge data associated with the applicable tests using their unique CPT codes. Only patients admitted to the PHM service who had at least one IM obtained were included in this calculation.

## RESULTS

During the study 9,963 patient encounters were reviewed. The baseline period was from September 5, 2021, to January 1, 2022. The intervention period was January 2, 2022, to January 1, 2023, and the postintervention period was January 2, 2023, to June 24th, 2023.

**Fig. 2.**
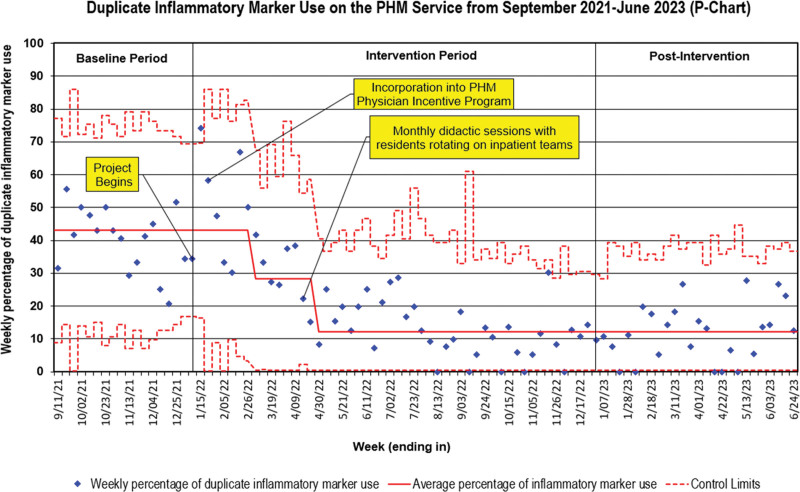
Statistical process control chart (p-chart) showing the primary outcome of weekly percentage of duplicate inflammatory marker use..

During the baseline period, 2,805 patients were admitted to the PHM service. Among those admitted, 19% had at least one IM resulted. The baseline duplicate IM use was 43% (Fig. 2). Within 2 months of the project initiation, a favorable downward shift was evident, with a noteworthy reduction in the duplicate IM use to 28%. The project’s introduction and the implementation of the PIP plan were two significant interventions that occurred before the first shift. Four months into the project, there was a notable second shift, with the percentage of duplicate IM use further dropping from 28% to 12%. This reduction was sustained throughout the remainder of the intervention and postintervention periods.

**Fig. 3.**
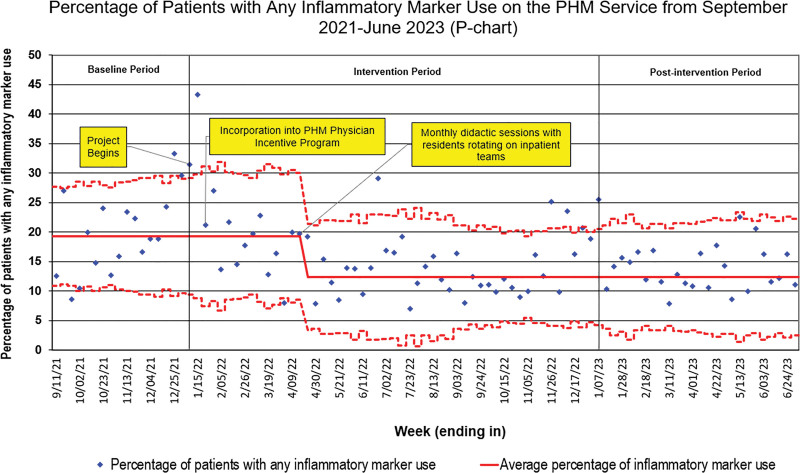
Statistical process control chart (p-chart) showing the secondary outcome of total inflammatory marker use.

The total percentage of any IM use on the PHM service also decreased from a baseline of 19% to 12% within 4 months of the start of the project (Fig. 3). The special cause variation from November 2022 to January 2023 was linked to an increased number of patients admitted to the hospital with respiratory illnesses. However, no specific cause was identified for the special cause variation observed in June 2022.

The process measure was the percentage of monthly education sessions provided to residents. Although monthly education was provided to residents starting in March 2022, the process was not initially monitored. However, from August 2022 to December 2022, the process was tracked, and hence, the data are only available for this time frame. A total of 10 resident education sessions were scheduled, with nine completed, resulting in a 90% completion rate.

**Fig. 4.**
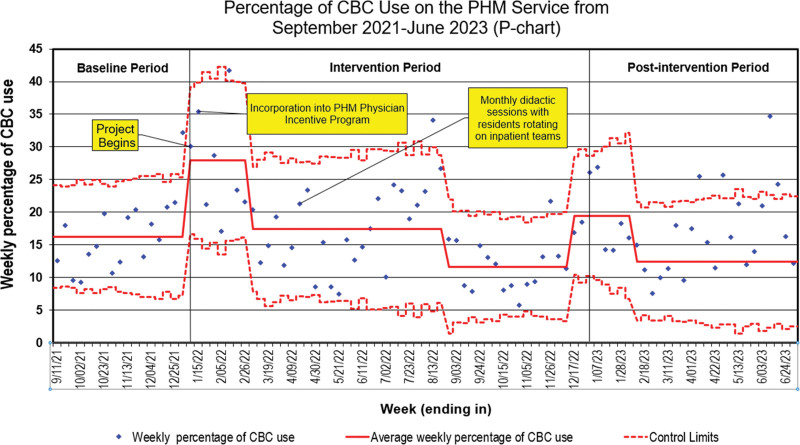
Statistical process control chart (p-chart) showing weekly CBC use.

The average LOS decreased slightly from 2.6 to 2.5 days (**Figure 1, Supplemental Digital Content 1,** http://links.lww.com/PQ9/A601). The 7-day readmission rate was 2.8%, which remained unchanged throughout the intervention period (**Figure 2, Supplemental Digital Content 1,** http://links.lww.com/PQ9/A601). The baseline CBC utilization was 14%. CBC used varied, peaking from November 2021 to February 2022, July to August 2022, and November 2022 to January 2023. At the end of the intervention period, CBC usage was 13%. Four months into the postintervention period, CBC usage increased to 21% (Fig. 4). There was special cause variation in CBC use, indicated by data points falling outside the control limits, mirroring the pattern observed in the overall utilization of IM.

Patient charges for IM testing were reduced by 9% from a mean of $175 per patient to $160 per patient, corresponding to an approximate $18,000 reduction in charges during the 12-month intervention period.

## DISCUSSION

Using the Institute for Healthcare Improvement model for improvement, duplicate IM use for patients admitted to the PHM service was decreased by 31%. In addition, the use of any IM for patients admitted to the PHM service was reduced by 7%, without increasing LOS or readmission rates. This is particularly important as healthcare costs continue to rise, and there is increasing pressure to provide high-quality care while managing costs effectively. CBC use varied, with no apparent correlation to trends seen in IM use. This likely reflects the many uses it has outside of infection.

Although several quality initiatives have focused on laboratory overutilization in the pediatric inpatient setting; we are unaware of any studies looking at reducing IM in children.^20,21^ Studies in adult inpatient settings have reported a successful reduction in duplicate IM using QI methodology and clinical decision support tools.^22,23^ Although we leveraged order sets, our study primarily focused on education, leaving ample opportunity to further enhance our approach through the utilization of clinical decision support tools.

In this project, the interventions with the most notable impact were the initiation of the project, the education sessions, and the addition of PIP. Engaging in discussions about the projects and placing an emphasis on high-value care potentially triggered the “Hawthorne effect,” a type of human behavior where individuals alter a particular aspect of their conduct upon realizing that they are being observed.^24^ Although the changes observed during the initial shift might be attributed to the Hawthorne effect, the subsequent shift appeared to be shaped by educational sessions, particularly those involving residents. Recognizing the pivotal role of residents as frontline providers in influencing laboratory orders, it is assumed that their involvement may have contributed to the observed shifts. Moreover, hospitalists were in the process of educating residents about high-value care. The inherent effect of teaching and fostering heightened awareness likely played a role in sustaining these shifts. It is important to note that the tracking of educational sessions commenced only in August 2022. Consequently, determining the precise extent of the impact becomes challenging, adding a layer of complexity to the analysis.

Financial incentives in pediatric medicine have shown varying levels of success in improving quality measures. For example, targeted financial rewards have been used to boost immunization rates among children, leading to modest yet notable improvement in vaccination completion.^25^ However, in another study, there was no significant impact on preventive care compliance, such as routine lead screening.^26^ Although it can be reasonably assumed that the observed changes in this project are attributable to the PIP plan, it is essential to note that this QI project constitutes only a small part of the overall PIP. This nuanced perspective emphasizes that, although the PIP may have played a role, other aspects contributed to the success achieved.

It is also important to be mindful of the natural variance within pediatric illness. During the baseline period, many patients were admitted to the PHM service for COVID-19 and multisystem inflammatory syndrome in children (MIS-C). The decrease in MIS-C prevalence likely accounted for some of the reduction in IM use in the early months of this project. However, duplicate ordering remained low despite a surge in pediatric illness and COVID-19 from November 2022 to January 2023. This occurred despite a high number of admissions for febrile infants and lower respiratory infections, anecdotally associated with frequent duplicate IM use at our institution. This sustained reduction in duplicate ordering and stable IM usage signifies a notable cultural shift and improvement in our healthcare practices.

### Limitations

This study had multiple limitations. This study was performed on patients admitted to a single academic medical center with an in-house laboratory with quick turnaround times, which may limit the generalizability of the work. At the project’s onset, there was a notable surge in the incidence of COVID-19 and MIS-C, which likely played a role in the increased use of IMs. Data on diagnosis-based IM utilization were not collected throughout the project lifecycle, making it challenging to pinpoint which diagnoses influenced the heightened use of IMs. Similarly, it is difficult to know the clinical and financial impact at the patient level as none of the measures were patient-oriented. For example, the economic impact was estimated using patient charges, which cannot be directly translated to patient savings as the rate negotiated by each insurance company will be different. We excluded IMs ordered in the ED, potentially underestimating the rate of IM use. Finally, there is concern for the long-term sustainability of the project as education and PIP have a low level of reliability. Although the impact may diminish with time, data from the 6-month postintervention period shows promising results.

## CONCLUSIONS

Duplicate IM and IM use was decreased at our institution without increasing LOS or readmission rates using QI methodology. Limiting overuse remains essential in high-value care, necessitating further studies to pinpoint interventions that effectively reduce overuse. Further studies should explore the environment- and physician-dependent factors influencing duplicate ordering. Moreover, the exact utility of PCAL in pediatrics remains an area worthy of exploration. Establishing guidelines for newer tests to reduce variation could contribute significantly to implementing best practices in healthcare.

## ACKNOWLEDGMENTS

The authors thank Dr. Michael T. Bigham and Ms. Rachel White for their assistance with this study.

## Supplementary Material


