# Supplementary material for: A Quality Improvement Initiative to Reduce Duplicate Inflammatory Marker Use
Source: Pediatr Qual Saf. 2024 Sep 19;9(5):e769. doi: 10.1097/pq9.0000000000000769 (PMC11412711; doi:10.1097/pq9.0000000000000769)
Supplement: Supplementary file 1 [file pqs-9-e769-s001.pdf]

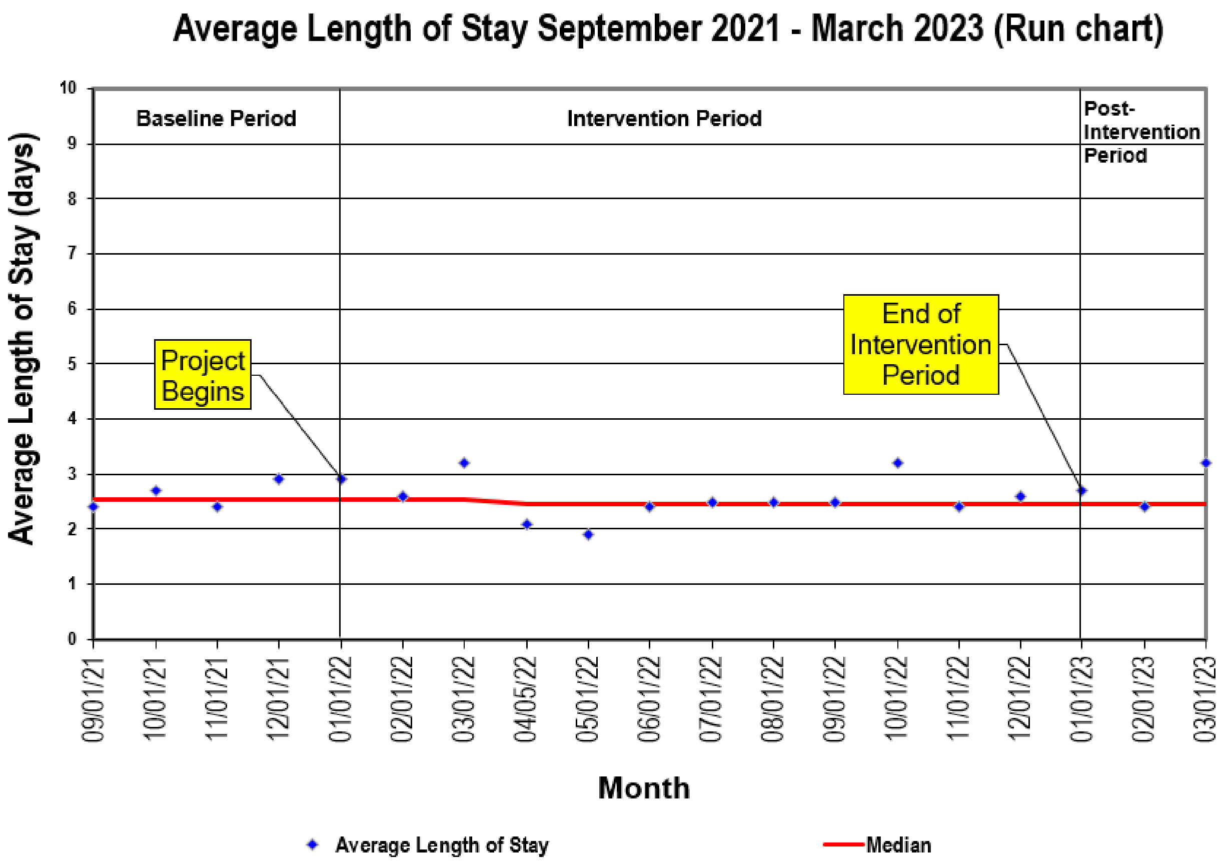

SDC, Figure 1. Run chart showing average length of stay.

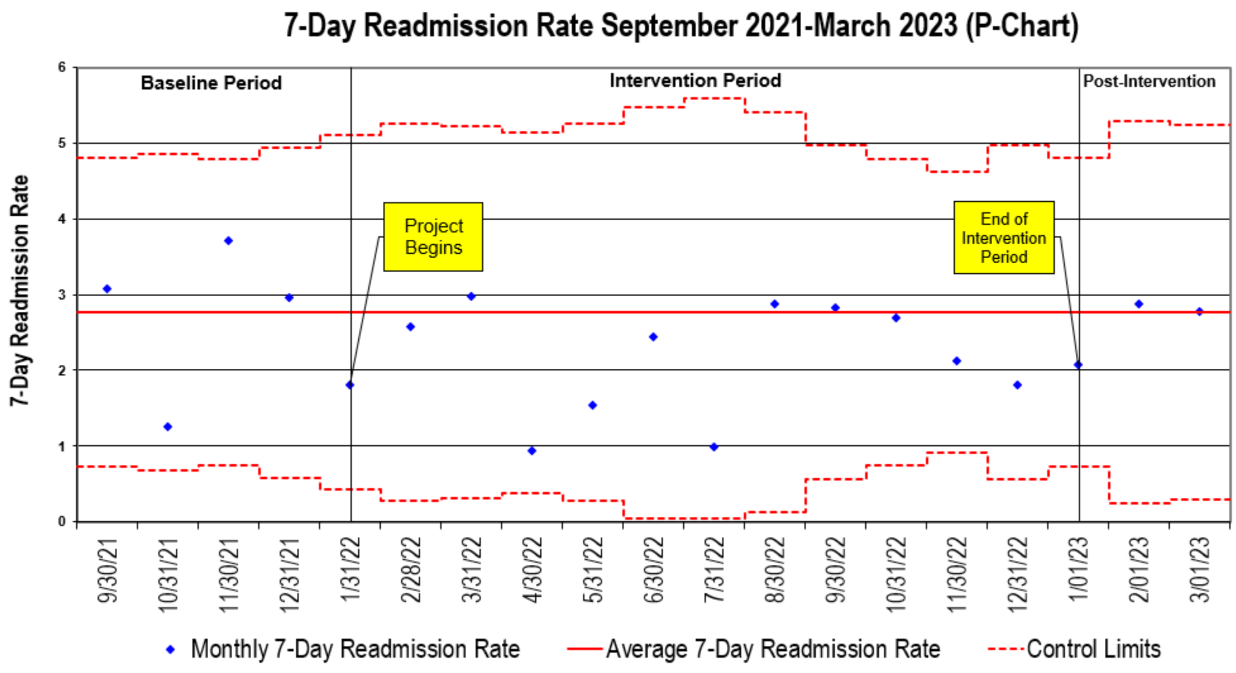

SDC, Figure 2. Statistical process control chart (p-chart) showing 7-day readmission rate.
